# CAR-engineered cell therapy in severe asthma: a target–function–patient framework for translational decision-making

**DOI:** 10.3389/fimmu.2026.1987098

**Published:** 2026-10-28

**Authors:** Jiang-ming Zhang, Xiao-long Ma, Xiang Zhang

**Affiliations:** 1 The First Hospital of Jiaxing, Affiliated Hospital of Jiaxing University, Jiaxing, Zhejiang, China; 2 School of Medicine, Shanghai University, Shanghai, China

**Keywords:** asthma, CAR-T, CAR-Treg, chimeric antigen receptor, eosinophils, immune tolerance, precision immunotherapy

## Abstract

Severe asthma is increasingly managed with endotype-guided biologics, yet a clinically important subgroup remains corticosteroid-dependent, repeatedly exacerbating, or unable to achieve durable multidomain remission. Chimeric antigen receptor (CAR)-engineered cells offer a qualitatively different therapeutic logic: recognition of a cellular compartment can be coupled to selective depletion, localized regulation, or payload delivery, potentially enabling finite immune reprogramming rather than continuous mediator blockade. This Review asks under what conditions that added biological risk could be justified in asthma. We classify evidence into four proximity tiers—direct asthma preclinical evidence, patient-derived ex vivo or disease-adjacent preclinical evidence, human clinical evidence in other immune-mediated diseases, and broader platform evidence—and use this hierarchy to support explicit go/no-go judgments rather than catalogue constructs. Direct asthma studies support lung-directed CAR-regulatory T cells, IL-5/IL-5Rα-directed cytotoxic strategies, multifunctional type 2 pathway control, and allergen-specific CAR-Tregs; clinical efficacy in asthma has not been established. We propose a target–function–patient framework organized around four mandatory gates: clinical necessity, mechanistic coherence, targetability, and reversibility planning. Targets should be prioritized by pathogenic necessity and physiological dispensability; the cellular chassis and receptor program should be chosen according to the required biological function; and treatment should be restricted to biomarker-defined patients whose residual disease burden justifies living-cell therapy. In asthma, maximal cytotoxicity and indefinite persistence are not default virtues. The most plausible initial role is a narrow, biologic-refractory population in whom a controllable, mechanistically verified intervention can durably interrupt a disease-maintaining cellular circuit while preserving host defense and tissue repair.

## 1 Introduction

Asthma is a heterogeneous chronic airway disease in which variable airflow obstruction, airway hyperresponsiveness, mucus dysfunction, epithelial injury, and inflammation arise from different combinations of immune and structural mechanisms. Mechanistic studies increasingly divide the clinical label into biologically distinct endotypes rather than a single inflammatory continuum (1–4). This shift has enabled precision treatment, particularly for type 2-high disease. Inhaled corticosteroid-containing regimens remain foundational, while severe asthma can now be treated with biologics directed against IgE, IL-5 or IL-5Rα, IL-4Rα-mediated IL-4/IL-13 signaling, and the epithelial alarmin thymic stromal lymphopoietin (TSLP). Randomized trials and contemporary reviews show substantial reductions in exacerbations and oral corticosteroid exposure in appropriately selected patients (4–8).

The success of biologics changes the question that a cell-therapy review must answer. The unmet need is no longer simply whether an inflammatory pathway can be inhibited, but why severe disease persists in some patients despite increasingly precise pathway suppression. Clinical remission is achievable yet incomplete and definition-dependent, and real-world analyses identify longstanding disease, fixed airflow limitation, corticosteroid dependence, obesity, and other comorbidities as barriers to remission (4, 9, 10). Repeated systemic corticosteroid exposure also carries cumulative toxicity (11). Any CAR-based intervention must therefore offer a qualitatively different benefit from maintenance biologics—for example, removal of a disease-maintaining cellular reservoir, restoration of tolerance, or durable interruption of a self-sustaining pathogenic circuit after a finite treatment.

CAR technology provides that possibility because antigen recognition can be linked to programmable cellular function. In oncology, engineered cells have demonstrated expansion, trafficking, persistence, and target-cell elimination; in autoimmune disease, CD19-directed CAR-T therapy has produced sustained remissions after withdrawal of conventional immunosuppressive therapy, findings consistent with, but not by themselves proof of, an immune-reset model (4, 12, 13). Transient *in vivo* CAR generation further shows that nonmalignant stromal populations can be targeted without permanent receptor expression (14). These observations do not establish asthma efficacy, but they show that CAR engineering can be adapted to nonmalignant disease.

Asthma-relevant CAR evidence has moved beyond pure speculation but differs in proximity to the disease. Direct asthma preclinical studies show that lung-directed CAR-Tregs can suppress experimental allergic airway inflammation (15), IL-5/IL-5Rα-directed CAR constructs can deplete eosinophilic populations (16), multifunctional IL-5-targeting CAR-T cells can combine cellular depletion with local interference in IL-4/IL-13 signaling (17), and allergen-specific CAR-Tregs can suppress birch-pollen allergic airway inflammation *in vivo* (18). Disease-adjacent engineering work shows that membrane IgE can serve as a cell-associated marker for redirecting T-cell recognition toward IgE-lineage cells (19), while patient-derived samples and preclinical hypereosinophilic-disease models support IL-5 CAR-T recognition of eosinophils and eosinophil precursors (20). Clinical observations after CAR-T therapy for other indications have also raised the possibility that profound immune remodeling may alter atopy and asthma, but these observations remain hypothesis-generating rather than evidence of asthma treatment efficacy (21).

Recent reviews span CAR technology across nonneoplastic diseases, precision treatment and emerging cell therapy in severe asthma, CAR-NK approaches in asthma, cell therapy for asthma more broadly, and CAR-T applications across respiratory diseases (4, 22–25). These syntheses are complementary but set a high bar for novelty. The present Review (**Table 1**) therefore does not primarily catalogue platforms or candidate targets. Instead, it treats CAR translation in asthma as a decision problem: when does the potential for finite immune reprogramming provide enough mechanistic advantage over effective biologics to justify the added risks of a living-cell intervention? We convert the fragmented evidence base into explicit go/no-go decisions linking pathogenic necessity, physiological dispensability, anatomical targetability, required engineered function, reversibility, and residual patient-level need. The aim is to distinguish targets that are technically addressable from those for which a cellular therapy could plausibly deliver a benefit that cannot be achieved more safely with a soluble therapy.

**Table 1:** Positioning of the present review relative to recent CAR/asthma reviews.

Review	Primary scope	Organizing logic	Distinct contribution of the present review
Zhou et al., 2026 (22)	CAR technology across nonneoplastic diseases	Broad survey of CAR platforms, targets, and disease areas beyond oncology	Asthma-specific evidence hierarchy; explicit comparison with effective biologics; mandatory translational entry gates
Chen et al., 2026 (23)	Individualized precision therapy for severe asthma	Places biologics and emerging cell therapies within the broader severe-asthma treatment landscape	Makes CAR translation the central question and links target necessity, engineered function, reversibility, and patient selection
Mohammad Taheri et al., 2024 (24)	CAR-NK approaches in asthma	Platform-specific discussion of CAR-NK design, proposed targets, manufacturing, and persistence	Chassis-agnostic framework that selects CAR-T, CAR-Treg, CAR-NK, or transient/regulated systems according to required function and risk
Zhou et al., 2026 (25)	Cell therapy for asthma	Broad cell-therapy review emphasizing MSCs, extracellular vesicles, immune-tolerance platforms, and translational barriers	Focuses specifically on CAR-engineered cells and converts evidence into target–function–patient go/no-go decisions
Han et al., 2025 (148)	CAR-T across respiratory diseases	Respiratory-disease survey spanning malignancy, asthma, infection, fibrosis, and autoimmunity	Asthma-specific, chassis-agnostic analysis benchmarked against modern severe-asthma biologics
Present Review	When CAR-engineered therapy could be justified over modern biologics in severe asthma	Four evidence-proximity tiers; four mandatory entry gates; domain-level target appraisal; first-in-asthma roadmap	Defines a narrow biomarker-selected clinical niche and an explicit decision framework for whether, how, and in whom to translate CAR therapy

### 1.1 Scope, literature identification, and evidence classification

This is a narrative translational review rather than a systematic review or efficacy meta-analysis. Literature was identified through targeted searches of PubMed from database inception through 31 August 2026. The search strategy combined disease terms, including asthma, severe asthma, and allergic asthma, with CAR-related and target-related terms including chimeric antigen receptor, CAR-T, CAR-Treg, CAR-NK, eosinophil, IL-5, IL-5Rα, IgE, allergen, autoimmune disease, transient CAR, mRNA CAR, and *in vivo* CAR. Because the relevant evidence spans asthma, allergy, autoimmune disease, and cell-engineering research, backward reference screening and forward citation chasing of key original reports and recent reviews were additionally used to identify studies not captured by asthma-specific searches. The regulatory status and clinical performance of comparator biologics were cross-checked against U.S. Food and Drug Administration and European Medicines Agency sources.

Studies were considered eligible when they provided information directly relevant to at least one component of the translational framework developed in this Review. These included: (i) original asthma-specific preclinical studies evaluating CAR-engineered cells; (ii) patient-derived ex vivo or disease-adjacent preclinical studies informing asthma-relevant target biology or targetability; (iii) human clinical studies of CAR-based therapies in other nonmalignant immune-mediated diseases that informed feasibility, durability, reversibility, or immune-reset potential; and (iv) mechanistic or platform studies directly informing antigen density, tissue trafficking, controllability, receptor design, or manufacturing. Studies without a clear connection to target selection, engineered-cell function, or translational decision-making were not prioritized for detailed discussion. Reviews and commentaries were used primarily to contextualize the field or identify primary studies rather than as evidence for target efficacy.

Studies were considered pivotal when they provided the most direct evidence within an evidence tier, introduced a distinct asthma-relevant target or engineered function, supplied patient-derived or human validation, or materially altered the assessment of feasibility, reversibility, or risk–benefit balance. Selection was therefore based on translational informativeness rather than positive outcome alone. Evidence was subsequently classified into four proximity tiers: direct asthma preclinical evidence; patient-derived ex vivo or disease-adjacent preclinical evidence; human clinical evidence in other immune-mediated diseases; and broader mechanistic or platform evidence.

Conference abstracts were included only when they provided otherwise unavailable and directly relevant emerging evidence. They are explicitly identified as conference abstracts and were treated as hypothesis-generating evidence rather than as equivalent to peer-reviewed full reports. Importantly, conference-abstract evidence was not permitted, by itself, to upgrade any domain-level judgment in **Table 2**. Because this was a narrative translational review, no quantitative pooling or formal risk-of-bias assessment was performed.

**Table 2:** Target-function matching and domain-level translational appraisal of candidate CAR strategies in asthma.

Pathogenic compartment	Target/platform-function	Asthma proximity	Evidence strength	Target fit	Control/reversibility	Comparator/finite benefit
Eosinophilic lineage	IL-5Rα/IL-5-based recognition; cytotoxic CAR-T or shorter-lived cytotoxic chassis	Direct asthma preclinical (16); patient-derived/disease-adjacent eosinophilic support (20)	Moderate — complementary preclinical and human-derived evidence; no asthma clinical data	Favorable — accessible lineage with clinically precedented depletion; downstream target	Moderate — short-lived chassis feasible; depletion may outlast CAR expression	High hurdle — effective anti-IL-5/IL-5Rα biologics; must show durable treatment-free benefit
Type 2 network	Cellular anchor + cytokine-modulatory payload; multifunctional/armored CAR	Direct asthma preclinical in established disease (17)	Moderate — therapeutic and rechallenge data, but essentially one platform/model family	Intermediate — addresses parallel type 2 pathways but broadens biological perturbation	Less favorable — added payloads and long-lived cells increase complexity/persistence	High hurdle — added cell-therapy risk must exceed established pathway-directed biologics
IgE-lineage B cells	Membrane IgE; mIgE-directed cytotoxic CAR	Disease-adjacent preclinical proof of concept (19)	Limited–moderate — targetability shown, but not in an asthma treatment model	Potentially favorable — cell-associated anchor; may miss long-lived IgE plasma cells	Moderate — transient designs possible; depletion depth/duration remains uncertain	High hurdle — must outperform anti-IgE therapy while preserving protective humoral immunity
Allergen-specific B cells	Antigen-display/CAAR for clone depletion or BAR-Treg for regulation	Indirect/disease-adjacent; not asthma-specific (4, 58, 149)	Limited — mechanistic proof of concept; heterogeneous functions/settings	Potentially favorable specificity; limited by polysensitization and epitope heterogeneity	Potentially favorable if clone-specific/transient; not validated in asthma	Potential unique benefit by sparing global B cells, but narrow coverage and unproven durability
Broad pathological B-cell compartment	CD19; conventional or transient CAR-T	Human clinical evidence in other autoimmune diseases; not asthma-specific (12, 13, 64)	Higher platform evidence, but low asthma-target-specific evidence	Unfavorable for most asthma — broad B-cell loss compromises protective immunity	Moderate–unfavorable — transient CAR limits exposure, but B-cell depletion may persist	Unfavorable — safer asthma biologics set a high bar for broad immune depletion
Long-lived IgE plasma cells	Plasma-cell antigen(s); ideally an IgE-selective plasma-cell strategy	Asthma-relevant rationale; no validated asthma-selective CAR target (4, 63)	Limited/discovery-stage — reservoir biology is relevant, but selective target definition is lacking	Currently unfavorable/undefined — broad plasma-cell targeting risks hypogammaglobulinemia	Unfavorable until an IgE-selective target and controllable exposure are available	High theoretical finite benefit, but current specificity and safety barriers dominate
Allergen-specific Th2/memory T cells	Allergen-specific peptide–MHC or related recognition; cytotoxic or regulatory system	Asthma-relevant mechanistic rationale and early recognition concepts (35, 36, 88)	Limited — strong rationale but little direct engineered-cell validation	Potentially specific; limited by HLA restriction, antigen heterogeneity, and no universal marker	Moderate in principle with regulatory/transient designs; irreversible depletion needs stringent validation	Potential durable antigen-specific memory modulation, but evidence is too limited for a favorable current risk–benefit judgment
Allergen-driven regulatory failure	Allergen-/tissue-directed CAR-Treg or CAlleR-Treg for localized regulation	Direct allergic-airway preclinical evidence (4, 15)	Moderate — two direct proof-of-concept studies; short/model-specific follow-up and no human asthma validation	Potentially favorable — regulation avoids obligatory target-cell destruction; antigen/context must be defined	Intermediate — noncytotoxic function is attractive, but Treg stability, persistence, and shutdown remain unresolved	Potentially distinct finite value through tolerance; durable benefit after cell contraction is unproven
Pathological remodeling fibroblasts	State-selective stromal target; preferably controllable CAR platform	Non-asthma fibrosis-model proof of concept (73, 74)	Moderate platform evidence; limited asthma-specific evidence	Currently unfavorable — normal repair functions and no validated asthma-selective antigen	Requires strong control/state gating; reversibility not validated in asthma	Could address remodeling unmet by biologics, but target-safety uncertainty currently dominates
Airway smooth-muscle pathological states	Undefined state-specific surface combination; exploratory logic-gated/regulatory strategy	Asthma biology relevant; CAR targeting remains conceptual (33, 74)	Limited/discovery-stage — no validated target or direct engineered-cell efficacy data	Unfavorable/undefined — essential physiology and insufficient state specificity	Speculative — logic-gated or regulatory rather than cytotoxic design would be required	Unmet structural disease is relevant, but no current basis for a favorable risk–benefit comparison

The five appraisal domains are reported independently and are not combined into a validated score or overall maturity category. Clinical-asthma proximity describes how directly the evidence informs asthma, whereas evidence strength/depth describes the robustness and breadth of the supporting data irrespective of disease proximity. Evidence-strength terms are qualitative: “higher” denotes substantial and/or human clinical evidence for the relevant platform or mechanism; “moderate” denotes direct or complementary preclinical support with important unresolved limitations; and “limited” denotes single-study, indirect, conceptual, or early proof-of-concept support. Target fit summarizes selectivity and physiological dispensability; control/reversibility summarizes the ability to limit exposure or terminate activity; and comparator/finite-treatment value indicates how difficult it would be to justify added cellular-therapy risk relative to existing asthma treatment and whether a finite intervention could offer a distinct advantage. These judgments are comparative decision aids rather than efficacy grades.

The included evidence was then interpreted within a translational decision framework that starts from a central tension: properties valued in cancer—maximal cytotoxicity, large expansion, and long persistence—can become liabilities when targets are physiologically useful cells and comparator therapies are effective and comparatively safe. We therefore use four mandatory entry gates: clinical necessity, mechanistic coherence, targetability, and reversibility planning (**Figure 1**). To avoid conflating product development with individual treatment eligibility, these domains are applied at two distinct levels. Program/product qualification asks whether the mechanistic hypothesis, target map, tissue access, and control strategy are adequate to justify testing the candidate. Patient eligibility asks whether an individual has sufficient residual need and whether the same target or pathogenic context is demonstrable in that patient. Only a candidate that passes program-level qualification should proceed to patient-level screening and target–function matching. Exposure, target engagement, pulmonary trafficking, host defense, tissue repair, and shutdown capacity are treated as linked pharmacological requirements. The preferred asthma product is therefore the least irreversible platform capable of breaking a validated pathogenic circuit, not necessarily the most potent or persistent one.

**Figure 1:**
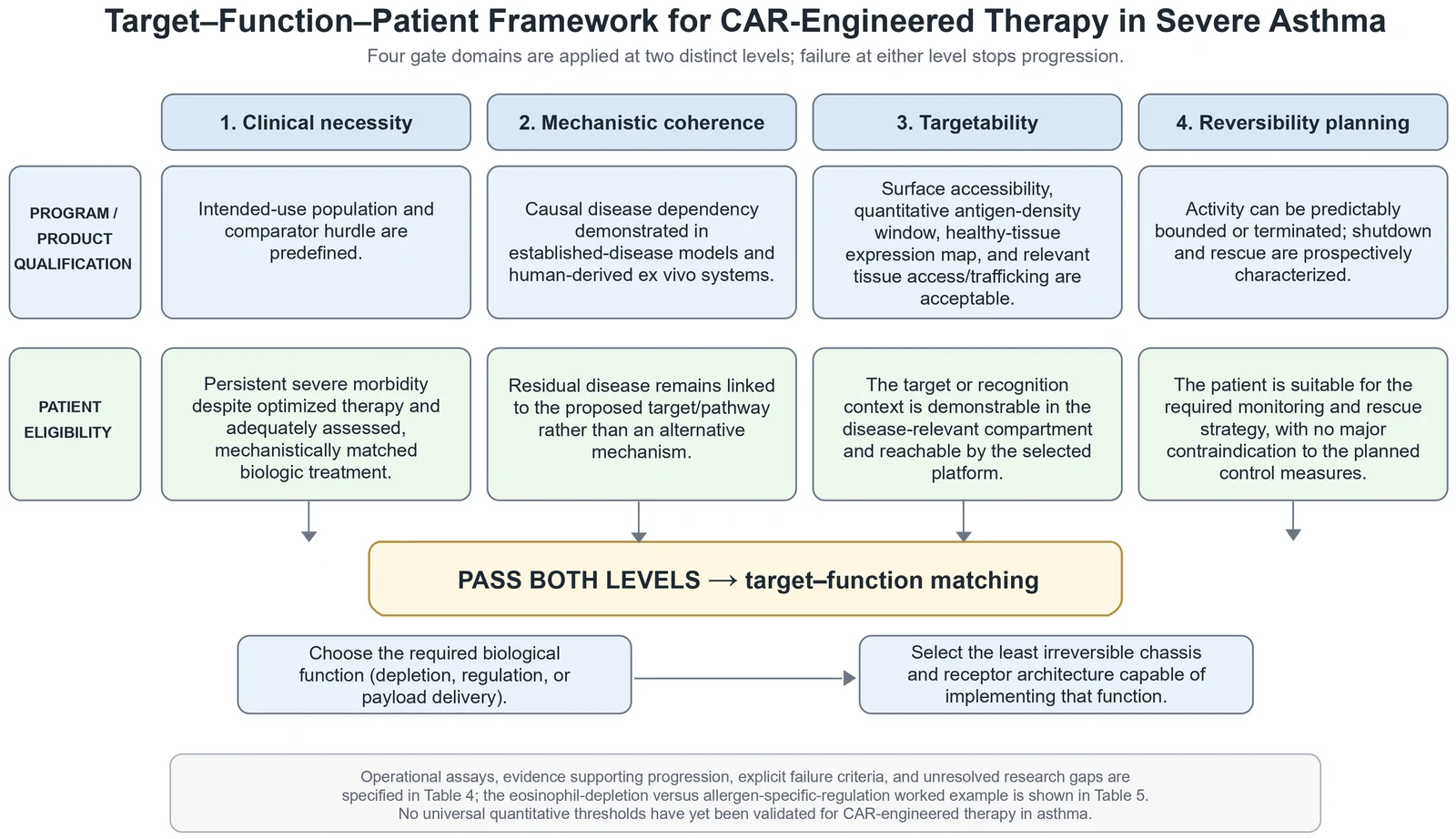
Target–function–patient framework for CAR-engineered therapy in severe asthma. The four gate domains are applied at two distinct levels. Program/product qualification establishes whether the intended clinical niche, mechanistic rationale, targetability, tissue access, and reversibility/control strategy are sufficient to justify clinical testing. Patient-level eligibility subsequently determines whether residual clinical need is sufficient and whether the relevant pathogenic mechanism and target or recognition context remain demonstrable in the individual, together with appropriate monitoring and rescue feasibility. Failure at either level constitutes a no-go for that candidate–patient pairing. Candidates that pass both levels proceed to target–function matching and selection of the least irreversible cellular chassis and receptor architecture capable of implementing the required biological function. Operational assays, evidence supporting progression, explicit failure criteria, and areas in which quantitative thresholds remain unvalidated are detailed in **Table 4**; **Table 5** provides a worked comparison of eosinophil depletion and allergen-specific regulation..

## 2 Biological rationale: where asthma may be vulnerable to cellular therapy

The biological case for CAR-engineered therapy is strongest when asthma is viewed as a network of interacting cellular states rather than a linear cytokine cascade. Type 2-high asthma typically involves epithelial alarmins, dendritic cells, Th2 cells, group 2 innate lymphoid cells (ILC2s), eosinophils, mast cells, basophils, and IgE-producing B-cell compartments, whereas type 2-low disease can include neutrophilic, Th17-associated, inflammasome-related, interferon-linked, metabolic, or paucigranulocytic states (4, 26–28). TSLP, IL-33, and IL-25 couple epithelial perturbation to type 2 immunity, and ILC2 biology provides an important route through which type 2 cytokine production can persist independently of conventional allergen-specific T-cell activation (4, 29, 30). This heterogeneity immediately argues against a universal asthma CAR: the same cell-surface target will not have equal causal importance across endotypes.

Type 2 inflammation is also redundant. IL-5 is critical for eosinophil biology, while IL-4 and IL-13 regulate IgE class switching, epithelial differentiation, mucus production, recruitment, and airway hyperresponsiveness. IgE, in turn, is the secreted output of a broader memory B-cell and plasma-cell program rather than an isolated soluble mediator (31). The therapeutic implication is that blocking an output and modifying its cellular source are not equivalent. A biologic can neutralize a cytokine or circulating immunoglobulin while treatment is maintained; a cell-based therapy could in principle remove, reprogram, or locally suppress the cell population capable of regenerating that output. The potential gain in durability is accompanied by a corresponding rise in biological risk because the same source population may also contribute to normal immune function.

Single-cell and spatial studies further weaken the idea that disease importance can be inferred from cell abundance alone. Airway tissue contains distinct epithelial, stromal, and immune states whose ligand–receptor interactions are reconfigured in asthma (32). Disease-driving activity can therefore be concentrated in a relatively small but strategically positioned cellular compartment. For CAR development, this suggests a target-prioritization rule: network centrality and causal dependence should be valued more highly than simple enrichment. A large population of downstream effector cells may be easy to detect and deplete yet rapidly repopulate if an upstream source or memory compartment remains intact. Conversely, targeting an upstream node may produce deeper control but create broader off-tissue or immunological consequences.

Structural cells add another layer. Airway remodeling includes epithelial barrier dysfunction, goblet-cell metaplasia, mucus plugging, extracellular-matrix deposition, fibroblast and myofibroblast activation, altered airway smooth-muscle mass, and vascular changes (4, 33, 34). These changes can become partly uncoupled from the inflammatory pathway that initiated them. That uncoupling matters because a therapy that eliminates eosinophilic inflammation may not reverse fixed structural disease, whereas a strategy directed against pathological stromal states could theoretically modify remodeling. At present, however, the safety threshold for structural-cell targeting in asthma is much higher than for targeting an abnormal malignant clone: epithelial cells, fibroblasts, and smooth-muscle cells are essential for tissue integrity and repair, and no asthma-specific surface antigen has yet been validated with the selectivity required for routine cytotoxic targeting.

Persistent immune memory provides the most compelling rationale for a finite cellular intervention. Tissue-resident memory T cells can remain in peripheral tissues and rapidly reactivate local inflammation after antigen re-exposure. Experimental allergic airway disease demonstrates nonredundant roles for circulating and lung-resident memory Th2 populations, including persistent local capacity to drive mucus metaplasia, airway hyperresponsiveness, and eosinophilic activation (35, 36). Human airway studies have also identified tissue-resident cytotoxic CD4+ states associated with severe disease (37). Beyond classical adaptive memory, epigenetic and metabolic reprogramming may create trained responses in innate and tissue-resident compartments (38). These observations support the idea that symptomatic control and eradication of the biological capacity for relapse are distinct goals.

Yet memory is precisely where indiscriminate targeting becomes dangerous. Pathogenic Th2 cells are defined largely by antigen specificity and state, not by a single lineage-exclusive surface molecule. Broad depletion of CD3+ or CD4+ cells would therefore be disproportionate for asthma. The same logic applies to B cells, eosinophils, mast cells, and stromal cells: disease association is not sufficient; the target must be both pathologically consequential and physiologically dispensable enough to tolerate depletion or reprogramming. Experience with on-target/off-tissue toxicity in other CAR settings and the growing use of multi-omic target discovery reinforce the need for comprehensive target-expression maps before clinical translation (39, 40).

The scarcity of disease-specific surface antigens is a major limitation for CAR-based therapy in asthma. Unlike malignant clones, many pathogenic compartments in asthma represent activated, differentiated, or remodeled states of otherwise normal immune or structural cells. Airway epithelial cells, fibroblasts, and smooth-muscle cells retain essential roles in barrier integrity, tissue repair, extracellular-matrix homeostasis, and airway function (4, 32–34), whereas pathogenic Th2 and tissue-resident memory T cells are defined largely by allergen specificity, tissue localization, and functional state rather than by a unique surface marker (35, 36). Consequently, targeting shared lineage antigens could eliminate physiologically important cells together with disease-driving populations, resulting in on-target/off-tissue toxicity and loss of protective immune or tissue functions. Target selection should therefore consider antigen density, expression stability, normal-tissue distribution, and physiological dispensability (39, 40). Where no sufficiently selective antigen exists, combinatorial recognition, transient CAR expression, or noncytotoxic regulatory strategies may be preferable to permanent cytotoxic depletion (4, 41–43).

We therefore propose four levels of CAR-addressable pathology. First, downstream effector populations such as eosinophils are attractive because their function is well defined and their depletion is clinically precedented. Second, source populations such as IgE-memory or antibody-producing compartments may offer greater durability by reducing the capacity to regenerate pathogenic mediators. Third, memory populations could interrupt recurrence at its origin but are difficult to identify selectively. Fourth, regulatory or structural nodes may be better approached through localized immunoregulation or state-dependent targeting than through permanent cytotoxic depletion. Moving upstream can increase the theoretical depth of disease modification while simultaneously increasing uncertainty and systemic risk.

The resulting target criterion is stricter than simply being ‘expressed in asthma’. A credible CAR target should be surface-accessible, enriched in a disease-maintaining population, sufficiently stable during the intended treatment window, scarce in essential healthy tissues, and linked to a therapeutic effect that cannot be achieved more safely with a soluble drug. Target validation must also account for anatomy. Blood expression is insufficient when the relevant population is maintained in airway mucosa, lung interstitium, lymphoid tissue, or bone marrow. This pathology-first view provides the foundation for the targetability gate in **Figure 1** and for matching engineered-cell platforms to disease mechanisms.

## 3 Engineering principles relevant to asthma

A CAR is a modular recognition-and-signaling system rather than a single therapeutic entity. Conventional constructs contain an extracellular recognition domain, a hinge or spacer, a transmembrane region, and intracellular signaling modules. Receptor architecture influences activation threshold, metabolic state, effector function, proliferation, and persistence (44–46). CD28- and 4-1BB-containing designs illustrate that costimulatory domains can produce distinct signaling kinetics and persistence programs rather than merely different degrees of ‘strength’ (47, 48). In asthma, these properties should be treated as tunable pharmacological variables rather than inherited from oncology designs.

The key engineering distinction is between recognition and function. A CAR defines what the cell senses; the chassis and intracellular program determine what it does after sensing. Conventional CAR-T cells typically use an antibody-derived binding domain to recognize a cell-surface antigen directly and independently of peptide–MHC presentation; in a cytotoxic T-cell chassis, this recognition is generally coupled to target-cell killing and depletion. CAR-Tregs instead aim to focus suppressive function at a defined antigen or anatomical site.

Antigen-display or chimeric autoantibody receptor (CAAR) systems operate differently: the extracellular domain contains the relevant antigen or autoantigen and functions as bait for a cognate B-cell receptor or cell-bound antibody. In a cytotoxic T-cell chassis, this architecture can selectively eliminate antigen-specific B cells, whereas antigen-displaying regulatory T cells are intended to suppress antigen-specific immune responses rather than deplete the recognized population. Peptide–MHC-based systems use a defined peptide–MHC complex as bait for cognate T-cell receptors and therefore recognize antigen-specific T-cell clonotypes rather than conventional lineage-associated surface antigens (4, 49). The CAlleR-Treg strategy represents another distinct recognition mechanism: the anti-Bet v 1 receptor senses the allergen in a context facilitated by allergen-specific antibodies and FcγR-expressing antigen-presenting cells, and this recognition activates a regulatory rather than cytotoxic program (50). Thus, receptor architecture determines what is sensed, whereas the cellular chassis and signaling program determine whether recognition produces depletion, regulation, or another engineered function. Regulatory-cell engineering is particularly relevant to chronic inflammatory disease because local suppression can theoretically interrupt multiple downstream pathways while preserving systemic immunity (4, 41–43). The challenge is maintaining lineage stability and avoiding tonic signaling or inflammatory conversion, making receptor design and manufacturing quality as important as antigen choice.

For CAR-Tregs, lineage stability and suppressive potency should therefore be demonstrated rather than inferred from cell identity alone. Lineage stability should be assessed by sustained expression of a regulatory phenotype, including FOXP3 with high CD25 and low CD127 expression, supported where feasible by maintenance of a stable Treg-associated epigenetic program such as demethylation of the FOXP3 Treg-specific demethylated region (4). Stability should also include the absence of acquisition of pro-inflammatory effector features, such as IFN-γ or IL-17 production, after repeated CAR stimulation or inflammatory challenge. Suppressive function should be evaluated in functional assays, preferably in the relevant antigen-dependent context, by reproducible inhibition of responder-cell proliferation and/or inflammatory cytokine production, with retention of activity after repeated stimulation. Because validated asthma-specific acceptance thresholds are not currently available, these parameters should be prespecified and reported quantitatively rather than assumed from phenotype alone (4, 51).

Alternative cellular chassis expand the design space. CAR-NK cells can provide cytotoxicity with different persistence and alloreactivity profiles and have shown clinical feasibility in hematologic malignancy (52, 53). An asthma-specific review has already surveyed CAR-NK design, proposed asthma targets, and emphasized potential advantages such as shorter persistence and off-the-shelf manufacture, while also underscoring the absence of asthma clinical validation (24). The present framework treats CAR-NK not as an intrinsically superior platform but as one option when bounded cytotoxicity, manufacturing scalability, and the target geography favor that chassis. Engineered macrophages demonstrate that CAR recognition can also be coupled to phagocytosis and myeloid functions (54). These platforms are not yet validated for asthma, but they are conceptually important. A short-lived cytotoxic chassis may be preferable when the therapeutic goal is bounded depletion, whereas a regulatory chassis may be preferable when the target is a tissue-associated antigen whose destruction would be unsafe. The appropriate comparison is therefore not ‘CAR-T versus no CAR-T, ‘ but which recognition–chassis–signaling combination best implements the desired disease intervention.

Persistence should be treated as a dose-duration variable rather than an intrinsic marker of product quality. Virus-free and mRNA-based approaches can produce transient CAR expression, allowing dose-by-dose control and limiting the consequences of unexpected target distribution (55). *In vivo* generation of CAR-expressing cells may also bypass conventional ex vivo manufacture and permit more programmable exposure (14). For asthma, transient platforms are especially valuable as target-validation tools: if short-lived CAR activity safely produces meaningful disease control, persistence can be increased only when recurrence shows that exposure—not target choice—is the limiting factor. This staged sequence is more defensible than beginning with a permanently persistent product in a nonmalignant disease.

Logic-gated and conditional systems can further improve specificity. Synthetic circuits can require combinations of antigens, implement sequential recognition, or modulate activity according to the local cellular context (4, 56, 57). Such approaches are relevant when no single asthma-associated marker is sufficiently selective. For example, a pathological stromal state might be defined by a combination of surface proteins rather than a unique antigen, while an immune population might require recognition of both lineage and activation-state markers. The trade-off is engineering complexity, which can increase manufacturing burden, validation requirements, and failure modes. Logic should therefore be introduced to solve a defined biological specificity problem, not simply because complex circuits are technically possible.

We therefore favor a function-first design sequence. The biological hypothesis should first specify whether disease control requires depletion, localized suppression, altered trafficking, niche remodeling, or local payload delivery. Only then should the cellular chassis and receptor architecture be selected. This avoids importing a familiar oncology construct and searching for an asthma antigen to fit it. In chronic airway disease, persistence, inflammatory output, and shutdown capacity should be designed as pharmacological variables around a validated pathogenic dependency.

## 4 Candidate targets and target-function matching

The most plausible CAR targets in asthma occupy different levels of the pathogenic hierarchy and therefore call for different engineered functions. **Table 2** summarizes target–function matching using five separate qualitative domains rather than a single overall maturity category: (1) clinical-asthma proximity, defined as how directly the cited evidence studies asthma; (2) evidence strength/depth, reflecting the breadth of supporting studies, therapeutic versus prophylactic design, duration of follow-up, and human-derived validation; (3) target selectivity and physiological dispensability; (4) feasibility of controlling or reversing engineered-cell activity; and (5) the comparator hurdle and plausibility of a finite-treatment advantage. These domains are intentionally nonweighted. Clinical-asthma proximity and evidence strength are kept separate because a body of evidence may be strong but disease-distant, or asthma-proximal but still limited to early preclinical proof of concept. No domain judgment indicates clinical readiness.

Pathogenic adaptive memory is an attractive upstream target because it could remove the reservoir that restarts disease after apparent remission. Lung-resident Th2 memory can persist and rapidly regenerate allergic airway pathology (35, 36). However, these cells lack a simple disease-specific surface antigen. Peptide–MHC or chimeric autoantibody/allergen-receptor concepts offer one route to antigen-specific recognition, potentially allowing deletion or regulation of clonotypes defined by allergen specificity rather than lineage markers (58). This approach is biologically elegant but difficult to generalize because asthma can be polysensitized, HLA-restricted, and driven by nonallergic mechanisms. The target is therefore upstream and potentially powerful, but the patient population would need unusually precise antigenic definition.

Antigen-specific recognition may limit collateral effects only when the recognition determinant is absent from essential healthy cells; specificity alone does not eliminate on-target/off-tissue risk. In polysensitized asthma, a single allergen may not account for clinically meaningful disease activity, and sensitization does not necessarily establish causal dominance (59). Epitope choice is recognition-system dependent: antibody-derived CARs require accessible antigenic determinants, peptide–MHC-based receptors depend on peptide presentation and HLA context, and allergen-directed CAR-Tregs may impose different recognition requirements. Epitope variability and potential cross-reactivity can further complicate target coverage (60). Multispecific receptor designs or localized CAR-Treg regulation offer possible alternatives, but the supporting multispecific engineering literature is largely outside heterogeneous allergic asthma, and clinical applicability remains unproven (61).

Humoral immunity offers several distinct intervention points. Membrane IgE provides a cell-associated target for eliminating IgE-lineage B cells while avoiding direct recognition of soluble IgE (19). Asthma is also associated with altered memory B-cell compartments (62), and chronic allergen exposure can generate long-lived IgE plasma cells in bone marrow, creating a persistent source of serological memory (4, 63). These observations explain why neutralizing circulating IgE and erasing the cellular capacity to regenerate IgE are mechanistically different. However, plasma cells create a difficult target problem: broadly expressed plasma-cell antigens could deplete protective antibody production, whereas an IgE-specific long-lived plasma-cell marker has not yet been established with sufficient selectivity.

Antigen-specific B-cell approaches may be safer than pan-B-cell depletion. Engineered antigen display or chimeric autoantibody receptor designs can exploit the B-cell receptor itself as a disease-specific recognition structure; however, the downstream effect is determined by the cellular chassis in which the antigen-display receptor is expressed. In cytotoxic CAAR-T cells, the displayed antigen functions as a bait for cognate B-cell receptors, enabling selective depletion of antigen-specific B cells. In contrast, antigen-displaying regulatory T cells use the same general recognition principle to promote antigen-specific immune suppression rather than target-cell killing (4, 49, 64). Thus, antigen-display strategies can mediate either selective depletion or targeted regulation, and these functions should be regarded as mechanistically distinct. Allergy studies further show that distinct memory B-cell states are poised to regenerate pathogenic IgE, reinforcing the concept that durable control may require intervention upstream of circulating antibody (4, 65, 66). For asthma, this suggests a future in which the therapeutic target is the allergen-specific clone rather than the entire CD19+ compartment.

Broad CD19-directed depletion provides the clearest clinical precedent that a finite CAR-T intervention can produce durable immune reprogramming in chronic immune-mediated disease, but it should not be imported into asthma without a high threshold. Preclinical lupus and clinical autoimmune studies demonstrate that CAR-mediated B-cell depletion can be deep and durable (64, 67). In a patient with severe biologic-refractory asthma and strong evidence that a pathological B-cell compartment is central, this experience could become relevant. For most asthma, however, the loss of broad B-cell immunity would be difficult to justify when safer biologics are available. CD19 should therefore be viewed as a benchmark for finite immune reprogramming, not as the default asthma target.

Eosinophils currently provide the most direct cytotoxic target. IL-5-anchored CAR-T cells depleted eosinophilic populations and prevented allergic airway inflammation in predominantly prophylactic HDM and OVA models, with protective effects reported for up to three months (16). A subsequent study moved substantially closer to a therapeutic setting: long-lived 5TIF4 cells combined eosinophil depletion with blockade of IL-4/IL-13 signaling and suppressed established OVA- and HDM-induced type 2-high asthma after a single infusion in immunocompetent mice without conditioning (17). In patient-derived blood and bone-marrow samples and preclinical hypereosinophilic-disease models, IL-5 CAR-T cells also recognized eosinophils and eosinophil precursors, supporting the feasibility of intercepting the lineage across developmental compartments rather than only in peripheral blood (20).

The appeal of eosinophil targeting is also its limitation. Eosinophils are downstream effectors, so their depletion may not erase allergen-specific memory, epithelial alarmin production, or other mechanisms that reconstruct type 2 inflammation. Multifunctional CARs can add cytokine modulation, but each added function broadens engineering complexity and the duration of immune perturbation. The clinical benchmark is high: benralizumab already produces profound eosinophil depletion, and twice-yearly depemokimab further reduces the practical burden of maintenance anti-IL-5 therapy (4, 68, 69). A CAR strategy therefore needs a demonstrable finite-treatment advantage, ideally with clinical benefit that persists after active CAR-mediated depletion has waned; reduced dosing frequency alone is not a persuasive value proposition.

A central theoretical limitation of cytotoxic CAR therapy in asthma is the possibility of prolonged or incompletely reversible biological effects. Asthma-directed preclinical studies demonstrate that eosinophil-lineage depletion can persist after a finite engineered-cell intervention (4). Unlike maintenance biologics, persistent cytotoxic CAR cells could continue to eliminate target cells without redosing. Even after receptor signaling is interrupted, recovery of an already-depleted immune-cell compartment cannot be assumed to occur immediately (4). Transient receptor expression, pharmacological control, and safety-switch systems can reduce further engineered-cell activity but do not guarantee reversal of its downstream effects (4). Target-cell recovery, host-defense function, and the time course of immune reconstitution should therefore be assessed prospectively; in the absence of clinical evidence for asthma-directed CAR therapy, the extent, duration, and reversibility of such effects in patients remain unknown.

Failure of an anti-IL-5/IL-5Rα biologic should therefore not itself be interpreted as a rationale for eosinophil-directed CAR therapy. The relevant question is why the biologic failed. If substantial target-positive eosinophilic lineage activity persists in airway, blood, or bone-marrow compartments despite verified and adequately dosed pathway-directed treatment, direct cellular recognition could in principle provide deeper or more durable depletion, particularly if residual precursor or tissue compartments remain accessible to engineered cells (4, 67). By contrast, if anti-IL-5/IL-5Rα therapy has already achieved effective eosinophil suppression while clinical disease persists, the residual pathology is more likely to reflect eosinophil-independent inflammatory pathways, comorbid disease, or structural remodeling. In that setting, increasing the depth or duration of eosinophil depletion would have little mechanistic justification. Demonstration of residual target-positive disease, rather than clinical nonresponse alone, should therefore be a prerequisite for an eosinophil-directed CAR program after biologic failure (4, 70).

Upstream epithelial alarmins and ILC2-centered networks are biologically important but less straightforward CAR targets. TSLP, IL-33, and related signals can orchestrate multiple arms of type 2 inflammation (71), and ILC2 function is shaped by cytokine, lipid, neural, metabolic, and environmental inputs (72). These pathways are attractive because they sit upstream of several effector processes, but the key molecules are often soluble mediators or expressed by physiologically important tissue cells. Direct cytotoxic targeting would therefore be difficult to justify. Engineered regulatory cells or locally delivered payloads may be more appropriate than depletion when the objective is to dampen an upstream network while preserving barrier function.

Structural-cell targeting is the most speculative strategy. FAP-directed engineered-cell approaches have reduced fibrosis in preclinical cardiac and pulmonary models (14, 73), and FAP-directed CAR-macrophages have shown activity in pulmonary fibrosis models (74). Asthma remodeling also involves disease-associated fibroblast, smooth-muscle, and epithelial states, but these cells participate in normal repair. Single-cell analyses reveal altered cellular communication during airway inflammation and remodeling (75), yet an asthma-specific stromal antigen with adequate selectivity has not been validated. Structural targeting should therefore follow state-specific surface-proteomic mapping and reversible preclinical perturbation rather than serve as a near-term clinical program.

CAR-Treg therapy offers a qualitatively different strategy because the therapeutic objective is regulation rather than deletion. Lung-directed CAR-Tregs can concentrate suppressive activity in pulmonary compartments and reduce airway hyperresponsiveness, eosinophilia, mucus, type 2 cytokines, and allergen-specific IgE (15). The CAlleR-Treg study extends this concept through a mechanistically distinct form of allergen recognition. In this system, anti-Bet v 1 CAlleR-Tregs respond to birch-pollen allergen in a context involving allergen-specific antibodies and FcγR-expressing antigen-presenting cells. Thus, the mechanism is not conventional CAR-mediated recognition and destruction of an antigen-bearing target cell; rather, FcγR-dependent presentation of antibody-associated allergen enables CAlleR engagement and activates a localized regulatory program (18). This approach may be especially valuable when a pathogenic antigen is known but the cells responding to that antigen are heterogeneous or difficult to eliminate safely.

Taken together, candidate targets can be ranked along two dimensions: pathogenic necessity and physiological dispensability. High-necessity, highly dispensable targets favor cytotoxic depletion; high-necessity but physiologically important targets favor regulatory, transient, or logic-gated intervention; low-necessity targets are unlikely to justify CAR therapy regardless of expression level. The domain-level view also clarifies why eosinophil targeting and CAR-Treg approaches should not be treated as equivalent simply because both previously occupied the highest relative category. Eosinophil targeting has a comparatively favorable target-definition profile and is supported by direct asthma plus patient-derived eosinophilic evidence, but its clinical comparator hurdle is unusually high because anti-IL-5/IL-5Rα biologics already achieve profound eosinophil depletion. CAR-Treg strategies offer a qualitatively different tolerance-restoring function and have direct allergic-airway proof of concept, but the current evidence is limited by short follow-up, model-specific antigen contexts, absence of human asthma validation, and unresolved Treg stability and persistence. More broadly, pathogenic necessity and physiological dispensability remain the mechanistic basis for function selection: highly dispensable disease-maintaining targets may justify depletion, whereas physiologically important compartments favor regulatory, transient, or logic-gated intervention. Candidate strategies should therefore be compared across the five domains rather than ranked by a single maturity label.

## 5 Evidence hierarchy: what has actually been demonstrated?

The direct asthma evidence base is small and should be interpreted according to both the engineered function and the timing of cell administration (**Table 3**). Importantly, prophylactic suppression of allergen-induced airway inflammation and treatment of established disease provide different levels of translational evidence. In the CEA-directed CAR-Treg study, cells were administered after systemic OVA sensitization but before completion of the airway allergen-challenge phase, and the intervention largely prevented the subsequent development of airway pathology (15). Similarly, IL-5-anchored CCAR-T cells were administered before allergen exposure in the acute HDM model, with subsequent experiments demonstrating protection lasting for up to three months (19). These studies establish proof of principle for tissue-focused immune regulation and eosinophil-lineage depletion, respectively, but they should not be interpreted as demonstrations that established asthma can be reversed.

**Table 3:** Experimental design and interpretive scope of direct asthma preclinical CAR studies.

Ref.	Receptor construct/cell product	Disease model	Timing of administration	Conditioning regimen	Principal endpoints	Follow-up and interpretation
Skuljec et al., 2017 (15)	CEA-specific CAR-Tregs; anti-CEA scFv with CD28–CD3ζ signaling	OVA-induced allergic airway inflammation in immunocompetent CEA-transgenic (CEAtg) mice	Predominantly prophylactic/preventive with respect to airway disease. CAR-Tregs were administered 7 days after initial sensitization but before completion of the intranasal allergen-challenge phase	No lymphodepleting conditioning reported	AHR; BALF inflammatory cells/eosinophilia; lung inflammation; mucus production; Th2 cytokines; allergen-specific IgE; pulmonary and lymph-node localization	Animals were analyzed at day 13, approximately 6 days after Treg transfer. Demonstrates tissue-directed regulatory activity, but not long-term disease modification
Chen et al., 2022 (16)	IL-5-anchored cytokine CAR-T cells (IL-5–CD28–CD3ζ), targeting IL-5Rα-expressing eosinophils and progenitors	HDM- and OVA-induced allergic eosinophilic airway inflammation in BALB/c mice	Prophylactic. In the acute HDM model, CAR-T cells were administered 1 week before the first HDM exposure; long-term experiments mainly assessed sustained protection rather than reversal of established disease	No lymphodepleting conditioning reported	Eosinophils in BALF, lung, blood, and bone marrow; BALF IL-5; inflammatory-cell burden; pulmonary histopathology	Protective effects were assessed for up to 3 months; supports prolonged prophylactic activity, not treatment of established asthma
Jin et al., 2024 (17)	Long-lived IL-5 CAR-T cells with BCOR/ZC3H12A depletion (5TIF); 5TIF4 additionally secretes an IL-4 mutein that blocks IL-4/IL-13 signaling	OVA- and HDM-induced type 2-high asthma, including established-disease and recurrence/rechallenge models	Therapeutic in established disease. Cells were administered after asthma induction in both OVA and HDM models; additional experiments assessed long-term control and recurrence	No conditioning regimen; experiments were performed in fully immunocompetent mice	Eosinophil depletion; lung inflammation; mucus/pathology; IL-13/type 2 signaling; IgE; airway physiology; CAR-T persistence	Long-term rechallenge experiments extended for months; this is the strongest of refs 15–18 for both treatment of established disease and sustained activity
Alcaraz-Serna et al., 2026 (18)	Bet v1-specific CAlleR 8 Tregs; anti-Bet v1 scFv linked to CD28 hinge/TM/signaling and CD3ζ	Birch-pollen extract-induced allergic airway inflammation in BALB/c mice	Both schedules tested. Prophylactic arm: Tregs on day 0 before allergen exposure. Treatment arm: Tregs on day 8 after four prior BPE exposures, followed by continued challenge	No lymphodepleting conditioning reported	BALF/lung eosinophilia; IL-10 and type 2 responses; mucus; IgE/IgG; respiratory mechanics/AHR; tissue localization	Treatment arm ended on day 15 (~7 days after transfer); prophylactic arm ended around day 14. Follow-up is too short to establish durable CAR-Treg persistence or durable remission

The enhanced activity of CEA-directed CAR-Tregs depended on CEA expression in transgenic recipient mice, which provided sustained antigen engagement within the pulmonary epithelium (15). This model therefore provides proof of principle that tissue-localized CAR activation can enhance Treg-mediated suppression, rather than validating CEA itself as a clinically relevant target for asthma. Translation of this strategy will require identification of an endogenous airway-associated antigen with appropriate tissue accessibility and sufficient selectivity to minimize on-target/off-tissue effects (39).

By contrast, the long-lived multifunctional 5TIF4 platform was tested therapeutically in established OVA- and HDM-induced type 2-high asthma in fully immunocompetent mice without a conditioning regimen (17). These cells combined eosinophil-lineage depletion with local interference in IL-4/IL-13 signaling and maintained disease control during long-term rechallenge. Among the current direct asthma studies, this provides the clearest evidence that an engineered-cell intervention can suppress already established experimental disease rather than merely prevent its development.

The long-lived 5TIF4 platform also illustrates why persistence-enhancing strategies require product-specific safety evaluation. Its prolonged persistence is achieved through disruption of BCOR and ZC3H12A rather than through CAR architecture alone. BCOR functions as a regulator of hematopoietic differentiation and has tumor-suppressive properties, with recurrent loss-of-function BCOR alterations identified in hematologic malignancies; experimental Bcor deficiency can enhance proliferative capacity and predispose to T-cell malignancy in mouse models (4). ZC3H12A (Regnase-1) is a critical negative regulator of immune activation through control of inflammatory transcripts, and T-cell-specific loss of Regnase-1 produces hyperactivated pathogenic T-cell phenotypes in experimental systems (76). These observations do not establish that 5TIF4 cells are oncogenic or autoimmune, as available preclinical studies did not demonstrate malignant transformation in evaluated models. However, combining enhanced persistence with disruption of endogenous regulatory pathways introduces product-specific uncertainties regarding clonal behavior, genome-editing safety, dysregulated activation, and long-term persistence. Such risks warrant dedicated evaluation before translation to nonmalignant diseases, and incorporation of independent elimination mechanisms may provide an additional safety layer (77).

The Bet v1-specific CAlleR-Treg study further broadened the evidence base by evaluating both prophylactic and treatment schedules (18). CAlleR Tregs suppressed birch-pollen-induced airway inflammation when administered before initial allergen exposure and also when transferred after repeated prior allergen exposures during an ongoing challenge protocol. However, the treatment experiment was assessed only approximately 7 days after cell infusion, and the prophylactic experiment approximately 14 days after infusion. These findings therefore support short-term regulatory activity but do not establish durable CAR-Treg persistence, durable tolerance, or sustained remission after engineered-cell activity declines. Longer follow-up incorporating serial cell tracking, withdrawal or contraction of engineered cells, and allergen rechallenge will be required before durability can be inferred (4, 42, 78).

Evidence from autoimmune disease is valuable because it tests whether a finite cellular intervention can reconfigure chronic immune pathology in humans. Early CD19 CAR-T experience and the subsequent CASTLE study support deep B-cell depletion and sustained clinical responses in treatment-refractory autoimmune disease (79). These data cannot be extrapolated directly to asthma because target biology, conditioning practices, organ risks, and acceptable toxicity differ. Their relevance is platform-level: they show that a living therapy can produce effects that outlast its conventional pharmacokinetic window when a disease-maintaining cellular compartment is correctly chosen.

Transient CAR therapy provides an equally important translational lesson. RNA CAR-T treatment in myasthenia gravis demonstrated clinical activity with a nonpermanent receptor platform, and a randomized placebo-controlled phase 2b study of BCMA-directed mRNA CAR-T therapy has now strengthened the clinical evidence for repeated transient cellular targeting in autoimmune disease (4, 76, 80). For asthma, this may be more informative than highly persistent oncology-style products because it shows that cellular therapy can be dosed as a controllable intervention rather than an irreversible one-time event. A transient platform could be particularly useful during early target validation or in patients for whom permanent lineage depletion would be unacceptable. Contemporary commentary has likewise highlighted the translational significance of bringing CAR-based immunotherapy into airway disease (81).

The evidence should therefore be read as a hierarchy rather than as a single body of efficacy data. Tier 1 comprises peer-reviewed direct asthma preclinical evidence (4, 15–17), with additional emerging conference-level direct evidence such as IgE-CAR-T depletion of FcϵRI-positive basophil and mast-cell populations (82). Tier 2 comprises patient-derived ex vivo or disease-adjacent preclinical evidence, including membrane-IgE engineering and IL-5/IL-5Rα targeting of patient-derived eosinophilic compartments (19, 20); related severe-atopy engineering concepts have also been reviewed (83), and conference-level IL-5 CAR-T data extend this disease-adjacent evidence across eosinophilic respiratory disorders (84). Tier 3 comprises human clinical evidence in other immune-mediated diseases, including durable clinical responses following B-cell-directed CAR-T therapy and repeated transient mRNA CAR pharmacology (4, 12, 13, 76, 79, 80, 85–87). Tier 4 comprises broader mechanistic and platform evidence informing target selection, trafficking, controllability, antigen-density discrimination, and product design (4, 14, 39, 40, 55–57, 88, 89). None of these tiers establishes clinical efficacy of CAR-engineered therapy in asthma; together they define which uncertainties must be resolved before first-in-asthma trials (4, 22–25).

## 6 Patient selection: applying the four gates

The threshold for CAR therapy in asthma must be substantially higher than the threshold for starting a biologic. Severe asthma should first be confirmed according to established definitions, with optimization of inhaled therapy, verification of adherence and inhaler technique, management of comorbidities, and removal of modifiable exposures (90). For the purposes of this Review, “biologic-refractory severe asthma” should not be defined simply by prior exposure to a biologic. Rather, it refers to persistent clinically important disease after a mechanistically appropriate biologic has been administered at the approved asthma dose and schedule, with treatment exposure verified and response assessed over an adequate interval. Current severe-asthma guidance recommends an initial biologic trial of at least approximately 4 months, with extension to 6–12 months when the response remains unclear. Response should be documented across multiple domains, including exacerbation frequency and severity, symptom control, lung function, systemic corticosteroid requirement or other treatment intensity, and, where relevant, biomarker and Type 2 comorbidity responses. Intolerance or a contraindication may justify earlier discontinuation but should be distinguished from mechanistic treatment failure (91, 92). This operationalizes the clinical-necessity gate in **Figure 1**: CAR therapy should be considered only after conventional precision treatment has been appropriately optimized and evaluated.

Importantly, biologic-refractory disease is not mechanistically uniform. For eosinophil-directed CAR therapy, at least two failure states must be separated. In one, clinically relevant target-positive eosinophilic lineage activity persists despite an adequate trial of anti-IL-5/IL-5Rα therapy (91, 93). Persistent eosinophils or eosinophil precursors in disease-relevant compartments could indicate incomplete suppression of the intended cellular target and provide a mechanistic rationale for testing whether direct cellular recognition can achieve deeper or more durable depletion. This remains a hypothesis that would require demonstration of residual target burden, target accessibility, and CAR-cell trafficking rather than being inferred from clinical nonresponse alone (16, 20). In the second state, eosinophils are effectively suppressed but substantial symptoms, exacerbations, or airflow limitation persist because disease is maintained by parallel inflammatory pathways, comorbid mechanisms, or relatively fixed airway remodeling (94). Further eosinophil depletion would be unlikely to address that biology. Such patients should therefore not be selected for eosinophil-directed CAR therapy merely because an anti-IL-5/IL-5Rα biologic has failed clinically (95).

The comparator is therefore not simply prior biologic use, but a documented biologic response phenotype. Licensed biologics already reduce severe exacerbations, corticosteroid exposure, and other clinically important outcomes, while some patients achieve multidomain remission (6, 96). The first plausible CAR population is consequently a narrow group with persistent severe morbidity after adequately dosed and adequately assessed trials of mechanistically matched biologics, together with evidence that the proposed cellular target remains present and causally relevant (4, 97).

An important patient-selection dilemma is that individuals with the greatest disease burden may also have limited pulmonary reserve, increasing their vulnerability to CAR-associated inflammatory complications. Although severe asthma may not intrinsically increase CAR-related toxicity, cytokine-release syndrome, hypoxemia, or pulmonary edema could have more serious consequences in patients with substantial airflow limitation (4, 98). Therefore, eligibility assessment should consider baseline lung function, exacerbation history, prior respiratory failure, and cardiopulmonary comorbidities alongside endotype and target confirmation. As no asthma-specific CAR eligibility threshold for FEV1 has been established, initial trials should prioritize clinically stable, biologic-refractory patients with adequate respiratory reserve, with cautious expansion to higher-risk populations as pulmonary safety data become available.

Endotype assignment must also become more rigorous than a single blood biomarker. Blood eosinophils, exhaled nitric oxide, IgE, and other markers are clinically useful, but they vary over time and can be modified by corticosteroids and biologics (99–101). For a therapy that may eliminate cells for months or years, the target itself should be measured whenever feasible. A candidate IL-5Rα-directed program should document persistent target-positive eosinophilic lineage activity; an IgE-lineage program should establish the relevant memory or antibody-producing compartment; an allergen-specific regulatory program should demonstrate a dominant and clinically meaningful allergen.

An important translational gap is the incomplete mapping between clinical asthma phenotypes and the cellular endotypes that CAR products would target. Labels such as eosinophilic, allergic, or type 2-low asthma support clinical stratification but do not identify a unique pathogenic cell population, confirm target stability or accessibility, or establish causal relevance. Patients with similar phenotypes may have distinct airway programs, while multiple inflammatory pathways may coexist in one patient (4, 102, 103). Therefore, clinical phenotype, prior biologic eligibility, or treatment failure cannot replace direct validation of a CAR-relevant target–function relationship. Until these mappings are prospectively validated, uncertain target identification remains a major implementation barrier and should preclude the proposed product–patient pairing.

Tissue-level profiling is likely to become necessary. Transcriptomic studies of sputum, epithelial brushings, and bronchial biopsies reveal molecular clusters that are not fully captured by conventional phenotyping, and multi-omic integration can distinguish severe neutrophilic and eosinophilic states (4, 102, 103). Single-cell and spatial lung atlases further show that target expression, cell state, and intercellular communication vary by anatomical niche (99, 100). This matters because a CAR cell encounters tissue antigen, not a blood biomarker. Patient selection should therefore evolve toward combined clinical severity, longitudinal biomarker behavior, and direct evidence that the proposed target is present in the disease-relevant compartment.

This challenge is particularly relevant in patients considered for CAR therapy, who may have received corticosteroids and multiple biologics targeting IgE, IL-5/IL-5Rα, IL-4Rα, or TSLP. These treatments can alter inflammatory-cell populations and biomarker levels, so blood, sputum, or airway samples may not reflect the original pathogenic endotype. Oral corticosteroid exposure has been associated with lower blood eosinophil counts and phenotype misclassification (104), while mepolizumab-treated patients may retain distinct inflammatory programs despite a shared type 2-high classification (105). Therefore, a negative biomarker during treatment does not exclude a historical endotype, whereas a historical biomarker does not confirm persistence of an actionable target. Patient selection should integrate treatment history, pretreatment data when available, serial biomarkers, and direct target or functional testing in relevant compartments. Therapy should not be withdrawn solely to reveal an endotype for experimental CAR selection.

Target density and normal-tissue distribution should be treated as eligibility variables rather than laboratory details. CAR activity can depend strongly on antigen density, and engineered circuits can be tuned to discriminate between high and low expression states (106, 107). Conversely, single-cell analyses of candidate antigens can reveal on-target/off-tissue expression that is not obvious from bulk datasets (108). A patient with high target density in a pathogenic compartment and negligible expression in essential normal tissue is fundamentally different from a patient with the same diagnostic label but a less favorable target map.

To make the gates usable in early translation, the same four domains should be read as a two-level decision system rather than as a single eligibility checklist. Clinical necessity is primarily a patient-level question. Mechanistic coherence and targetability require program-level evidence that the proposed intervention can alter a disease-maintaining compartment safely and patient-level evidence that the same compartment remains relevant and accessible in the individual. Reversibility planning is primarily a product- and protocol-level requirement, although a patient must also be suitable for the proposed monitoring and rescue strategy. **Table 4** specifies candidate assays, evidence that would support progression, and explicit failure criteria. These criteria are proposed translational decision rules, not validated clinical cutoffs.

**Table 4:** Operationalization of the four translational gates for asthma CAR development and early-trial eligibility.

Gate	Application level	Candidate assays/minimum evidence set	Evidence supporting progression	Failure/no-go and validation status
1. Clinical necessity	Primarily patient level	Confirm severe asthma after optimized inhaled therapy; verify adherence and inhaler technique; document exacerbations and systemic corticosteroid exposure; measure symptom control (e.g., ACT/ACQ) and spirometry; verify the dose, schedule, duration, and multidomain response to mechanistically matched biologic therapy (4, 90, 91).	Persistent clinically important morbidity after an adequately dosed and adequately assessed matched biologic, with residual disease burden sufficient to justify living-cell risk.	No-go if poor adherence, modifiable exposure, comorbidity, or inadequately tested standard therapy plausibly explains the burden, or if disease is adequately controlled. The duration of biologic assessment has guideline support, but no validated CAR-specific severity threshold exists.
2. Mechanistic coherence	Program level + patient level	Program: functional perturbation in established-disease models and human-derived ex vivo systems. Patient: candidate-specific evidence of pathway activity. For eosinophil-directed programs, this may include blood differential counts, induced-sputum eosinophils, and flow-cytometric assessment of the target on residual eosinophils/precursors; marrow assessment should be reserved for programs in which precursor targeting is integral and clinically justified. For allergen-specific programs, use exposure history plus skin-prick testing and/or serum allergen-specific IgE/component-resolved diagnostics, with ex vivo allergen-dependent activation or suppression assays where feasible (4, 99, 102, 103, 150).	A causal or strongly convergent link between the candidate compartment/function and disease, plus evidence that the same pathway remains active in the individual despite prior treatment.	No-go if the candidate is only associated with disease but not functionally necessary, or if the target pathway is already effectively suppressed while clinically important disease persists. No validated minimum “pathway contribution” or biomarker cutoff exists for CAR selection.
3. Targetability	Program level + patient level	Quantitative flow cytometry (ideally calibrated to estimate antigen molecules per cell) or an equivalent antigen-density assay; primary-cell CAR activation/killing/suppression across a target-density range; single-cell/spatial or surface-proteomic mapping of diseased and healthy tissues; and *in vivo* biodistribution/trafficking studies. Patient testing should confirm the target or recognition context in the disease-relevant compartment (4, 99–101, 151).	Surface-accessible target/recognition context at a construct-compatible density, acceptable expression in essential healthy tissues, and demonstrated access of the selected cell platform to the relevant anatomical compartment.	No-go if the target is absent, too sparse or heterogeneous, expressed at unsafe levels in essential healthy tissue, or anatomically inaccessible. Antigen-density and trafficking thresholds are construct-specific and have not been validated in asthma.
4. Reversibility planning	Primarily product/protocol level; patient rescue feasibility	*In vitro* and *in vivo* time-course testing of transient expression, pharmacological on/off control, suicide-switch or adaptor shutdown where applicable; time-to-shutdown and recovery; cell persistence and immune-reconstitution monitoring; and prespecified clinical stopping, rescue, and re-treatment procedures (4, 55, 76, 77, 122).	Engineered activity can be predictably bounded or terminated on a clinically relevant timescale, with predefined rescue options and a monitoring plan capable of detecting pulmonary or systemic toxicity early.	No-go if activity cannot be terminated or meaningfully attenuated, if expected target-cell loss cannot recover to an acceptable state, or if rescue is impractical for the intended population. No validated asthma-specific maximum persistence or shutdown-time threshold exists.

### 6.1 Operationalizing the four gates: product qualification versus patient eligibility

For each gate, assays should be chosen to test the specific failure mode that would invalidate translation. The examples below are intended as a minimum evidence set for early development. They deliberately avoid universal numerical thresholds where none have been validated in asthma.

The absence of validated numerical cutoffs is itself a research gap. Current evidence does not define a universal antigen-density threshold, minimum tissue target burden, minimum ex vivo response, acceptable duration of engineered-cell persistence, or maximum shutdown time for asthma. These parameters should therefore be prospectively calibrated for each construct using exposure–response, antigen-density, trafficking, and shutdown data in preclinical studies and then reported quantitatively in first-in-asthma trials rather than imported from oncology.

### 6.2 Worked example: eosinophil depletion versus allergen-specific regulation

A direct comparison illustrates that the gates discriminate between candidate mechanisms rather than simply identify “severe” patients. Eosinophil depletion and allergen-specific regulation can both be plausible in type 2-high asthma, but the evidence required to pass each gate is different (**Table 5**).

**Table 5:** Worked application of the four gates to two mechanistically distinct asthma CAR strategies.

Gate	Eosinophil-depleting CAR strategy	Allergen-specific regulatory strategy	How the gate discriminates
1. Clinical necessity	Severe eosinophilic asthma with persistent morbidity after optimized therapy and an adequate trial of mechanistically matched anti-IL-5/IL-5Rα therapy.	Severe allergic asthma with persistent morbidity after optimized therapy and appropriate biologic treatment, with a clinically dominant allergen for which antigen-specific regulation is biologically meaningful.	Both may pass on severity, but the comparator history must match the proposed mechanism.
2. Mechanistic coherence	Pass only if clinically relevant eosinophils/eosinophil precursors or related lineage activity persists despite anti-IL-5/IL-5Rα treatment. If eosinophils are already effectively suppressed while disease persists, this gate fails (4, 16, 20, 91, 92, 94, 95).	Pass only if a defined allergen remains a major disease driver, supported by concordant exposure history and sensitization (skin-prick and/or serum allergen-specific IgE, preferably component-resolved where informative), with ex vivo allergen-dependent activation/suppression supporting functional relevance when feasible. Polysensitized, nonallergic, or poorly attributable disease weakens or fails this gate (18).	Clinical nonresponse alone is insufficient: the eosinophil strategy requires residual target-positive disease, whereas the regulatory strategy requires demonstrable antigen dependence.
3. Targetability	Quantify residual eosinophilic lineage burden using blood and, where informative, induced sputum; measure the CAR-recognized target on residual eosinophils/precursors by flow cytometry; demonstrate construct-compatible antigen density and systemic access to tissue and/or marrow compartments; exclude unsafe normal-tissue activation.	Demonstrate that the engineered Treg is reproducibly activated by the relevant allergen in the required antibody/APC context, retains suppressive function in ex vivo assays, and can reach the airway compartment; a fixed target-cell antigen is not required (18).	Eosinophil depletion has a more conventional cell-surface targetability problem; allergen-specific regulation depends on a reproducible recognition context and tissue localization.
4. Reversibility planning	Prefer bounded cytotoxic exposure (for example, transient or shorter-lived chassis and/or a validated shutdown mechanism) because depletion may outlast receptor expression.	Require evidence of Treg lineage stability, bounded persistence, and a means to terminate or limit activity if generalized immunosuppression or phenotypic instability emerges (4, 42, 77, 78, 114).	The key failure mode differs: prolonged lineage depletion for the cytotoxic strategy versus unstable or uncontrollable regulatory function for the Treg strategy.
Current decision	Potentially testable only in a narrow residual-target-positive phenotype; comparator hurdle is very high because anti-IL-5/IL-5Rα biologics already achieve profound depletion.	Mechanistically distinct and potentially tolerance-restoring, but currently limited by allergen/context specificity, short preclinical follow-up, absent human asthma validation, and unresolved stability/persistence thresholds.	The gates do not rank both candidates as equally mature: they identify different eligible populations and different reasons for no-go.

This comparison also clarifies the meaning of a gate failure. A patient who fails the eosinophil-depletion pathway because eosinophils are already suppressed is not automatically ineligible for all engineered-cell approaches; the same patient could still be considered for a different mechanism only if that alternative independently passes mechanistic-coherence and targetability testing. Conversely, a patient with marked eosinophilia but no dominant allergen may remain a plausible depletion candidate while failing an allergen-specific regulatory program. The framework therefore selects a mechanism–product–patient pairing rather than severity alone.

## 7 Asthma- and lung-specific translational challenges

Pulmonary trafficking and compartment-specific exposure cannot be assumed from oncology experience. Asthma pathology is distributed across distinct anatomical niches, including the airway mucosa, lung interstitium, lymphoid tissues, and, for some targets, bone marrow. Lung-resident immune cells also occupy specialized airway and parenchymal niches regulated by tissue-homing programs such as CXCR6 (4, 106). Thus, systemic expansion or persistence of engineered cells does not necessarily indicate adequate exposure within the disease-relevant airway compartment, whereas excessive pulmonary accumulation could provoke local inflammation. Trafficking should therefore be assessed directly through biodistribution, tissue retention, target engagement, and pulmonary safety rather than inferred from peripheral blood kinetics.

The airway microenvironment may alter cellular function after arrival. Asthmatic eosinophils can modify extracellular-matrix programs in airway smooth-muscle cells and fibroblasts, while chemokine networks can bridge type 1 and type 2 inflammatory states (4, 109). Remodeling also changes extracellular matrix and tissue mechanics (110). These features could modify activation thresholds, cell motility, nutrient availability, and persistence. Preclinical models should therefore include established disease and remodeled airway tissue rather than testing only prophylactic treatment in acute allergen-challenge systems.

Local delivery may improve anatomical exposure but does not inherently guarantee efficacy or safety. Although regional CAR-T delivery and inhaled pulmonary engineering approaches are feasible (111, 112), deposition in asthmatic airways may be heterogeneous because of airway narrowing, mucus, altered airflow, mucociliary clearance, and anatomical variability. The optimal route also depends on target geography: airway-resident targets may favor local administration, whereas targets in lymph nodes, interstitium, or bone marrow may require systemic access. Local delivery should therefore be treated as a route-specific pharmacological strategy, with preclinical studies directly evaluating regional deposition, persistence, target engagement, efficacy, and pulmonary toxicity.

Spatial and temporal heterogeneity is another challenge. Severe asthma can shift under corticosteroids, infection, season, biologic therapy, and environmental exposure. A target that is abundant during an exacerbation may be sparse during stable disease, and different airway regions can contain different cellular ecosystems (113). Persistent CAR cells may therefore encounter a changing antigen landscape over time. Serial target assessment, or controllable systems that can be activated only when needed, may be particularly important in a disease whose biology is dynamic rather than clonally stable.

Prior treatment complicates both sampling and assay interpretation. Prolonged immunomodulation may reduce detectable target-positive cells, while peripheral-blood findings may not reflect residual airway or lung populations. Sputum and bronchoscopic sampling can also be burdensome and may not capture spatially restricted disease. Therefore, studies should prespecify sampling adequacy, treatment timing, cross-compartment concordance, and criteria for indeterminate results. Negative or low-yield samples should not exclude a previously present endotype, but presumed target persistence requires independent confirmation before irreversible targeting (114, 115).

The lung also has little tolerance for treatment-induced inflammation. Even transient cytokine release or localized edema may be clinically important in patients with limited respiratory reserve. Asthma-specific development should therefore prioritize low-inflammatory activation profiles, early pulmonary monitoring, and explicit stopping rules. The objective is not simply to deliver engineered cells to the lung, but to achieve the required anatomical exposure without converting therapeutic immune activation into a new cause of airflow obstruction.

## 8 Safety and therapeutic control

CAR-associated toxicity is well characterized in oncology, including cytokine release syndrome, immune effector cell-associated neurotoxicity, cytopenias, infections, and delayed complications (98, 116). Newer analyses emphasize that noncanonical toxicities and late morbidity can also define clinical risk (117, 118). Asthma changes the acceptable balance: because most patients can be managed with inhaled therapy or biologics and the disease is nonmalignant, a toxicity profile tolerated in refractory leukemia would be unacceptable. Early asthma studies should therefore be designed around prevention, reversibility, and minimum effective exposure rather than management of expected severe toxicity.

Although there is no *a priori* reason to assume that CAR-associated toxicities are intrinsically more frequent in severe asthma than in other indications, their pulmonary consequences may be particularly serious in patients with impaired respiratory reserve. Cytokine-release syndrome can cause hypoxemia and pulmonary edema, while treatment-associated infections or inflammation may further compromise respiratory function (4, 98, 116). Even modest respiratory deterioration could precipitate respiratory failure in patients with severe airflow limitation. This creates a clinical dilemma, as patients with the greatest unmet therapeutic need may also be least able to tolerate treatment-related complications.

Accordingly, first-in-asthma trials should incorporate careful assessment of baseline lung function, multidisciplinary risk evaluation, conservative dose escalation, and close monitoring of respiratory status. Minimizing lymphodepleting conditioning, establishing predefined intervention thresholds, and ensuring timely access to supportive care and CAR-cell shutdown mechanisms may further mitigate risk (4, 98). Although transient or switchable CAR platforms may improve treatment controllability, they cannot necessarily reverse established pulmonary injury (4). These considerations underscore the need to balance therapeutic benefit against individual respiratory vulnerability when selecting candidates for CAR-based therapy.

On-target/off-tissue toxicity is especially important because candidate asthma targets often belong to normal immune or structural lineages. Deep depletion of eosinophils, B cells, plasma cells, or stromal states may produce consequences that become apparent only after infection, vaccination, tissue injury, or long follow-up. The target-screening process should combine normal-tissue atlases, primary-cell testing, quantitative antigen-density measurements, and functional assays under inflammatory conditions. The safety question is not whether the antigen is absent from every normal cell; it is whether the level and context of normal expression are below the activation threshold of the engineered product (101).

Host defense is a major concern. Infection after CAR-T therapy reflects multiple interacting factors, including prior immunosuppression, lymphodepletion, B-cell or plasma-cell loss, cytopenias, and immune reconstitution (115). Long-term follow-up in oncology also highlights delayed complications that are not captured by the first weeks of observation (114). Reports of secondary malignancies after commercial CAR-T therapy reinforce the need for long surveillance, even though the absolute causal contribution of the engineered cells remains an area of active evaluation (119, 120). An asthma product that requires intensive lymphodepletion would therefore face a particularly difficult risk–benefit argument.

Alternative chassis may mitigate some risks. Allogeneic CAR-NK products have shown encouraging safety and efficacy profiles in oncology, with different persistence and cytokine characteristics from conventional autologous CAR-T cells (121). Whether those properties translate into safer depletion of an asthma-associated lineage remains unknown, but shorter persistence can be an advantage when irreversible activity is not required. CAR-Tregs may reduce inflammatory toxicity through a regulatory rather than cytotoxic mechanism, although their safety depends on stable suppressive identity and avoidance of excessive generalized immunosuppression (78).

Control systems should be considered a core design requirement, but controllability should not be equated with biological reversibility. Dasatinib can act as a pharmacological on/off switch for CAR-T signaling, while suicide switches and switchable adaptor systems provide routes to attenuate or terminate ongoing engineered-cell activity (4, 77, 122). These approaches can limit additional exposure after a toxicity signal emerges, but they cannot undo biological effects that have already occurred (77). Terminating CAR activity does not restore eosinophils, B cells, plasma cells, or other target populations that have already been depleted; it does not immediately reconstitute vaccine responsiveness or antimicrobial immunity; and it cannot repair established structural remodeling. Reversibility planning should therefore distinguish product controllability, defined as the ability to attenuate or terminate engineered-cell activity, from consequence reversibility, defined as the extent and time course over which depleted cell populations, protective immunity, and tissue function recover after activity has ceased. Both dimensions should be evaluated prospectively (4, 119). A shutdown mechanism should therefore be regarded as a means of preventing further injury rather than evidence that prior biological effects are themselves reversible.

This distinction is particularly relevant when CAR-mediated depletion is compared with established asthma biologics. Most biologics require repeated administration and can be withheld if adverse effects occur; circulating drug exposure subsequently declines, although pharmacodynamic effects—including eosinophil depletion after anti-IL-5Rα treatment—may persist beyond the final dose. Persistent CAR cells, in contrast, may continue to engage targets without redosing, and profound depletion of a physiological cell lineage may outlast CAR-cell elimination. Neither approach is instantly reversible, but the duration and predictability of immune reconstitution may be less controllable after a durable cytotoxic CAR intervention. Thus, prolonged biological effects are a potential limitation as well as a proposed advantage, and any benefit of treatment-free disease control must be weighed against persistent immune perturbation and delayed recovery of target-cell or protective immune functions.

Transient expression provides an additional layer of controllability by intrinsically limiting the duration of receptor activity, but transient CAR expression should likewise not be interpreted as guaranteeing reversal of downstream biological effects. Randomized clinical experience with mRNA CAR-T therapy in myasthenia gravis indicates that meaningful immune modulation does not necessarily require permanent receptor expression (76). In asthma, repeated transient dosing could define the minimum exposure needed to interrupt disease, with longer persistence considered only if recurrence demonstrates that the target is valid but exposure is insufficient. This creates a staged path from reversible perturbation to durable intervention.

An asthma-specific safety objective can therefore be stated simply: disease modification should not create a second chronic immune disorder. This implies preservation of vaccine responsiveness and antimicrobial defense where possible, avoidance of broad lineage depletion unless strongly justified, respiratory monitoring adapted to baseline lung function, and long-term assessment of immune reconstitution. Safety is not a downstream consideration in asthma CAR development; it helps determine which targets, functions, and platforms are acceptable in the first place.

## 9 Manufacturing and first-in-asthma clinical translation

Conventional autologous CAR-T manufacture is complex, individualized, and sensitive to cell quality, culture conditions, vector production, and release testing, all of which influence potency, consistency, and scalability (123–125). Rapid-manufacturing and point-of-care approaches could reduce treatment delay and prolonged ex vivo manipulation (123, 124). In asthma, these strategies may be particularly relevant to autologous CAR-Treg or other regulatory products requiring patient-specific target confirmation and preservation of lineage stability. However, decentralized manufacture would still require standardized release criteria, including viability, CAR expression, sterility, functional potency, and, for CAR-Tregs, maintenance of regulatory phenotype. Given the convenience of current outpatient biologics, point-of-care cell therapy would need to provide substantial durability or disease-modifying benefit to justify this added complexity.

Allogeneic “off-the-shelf” platforms could instead provide standardized products with greater availability and shorter treatment delays, but introduce risks of graft-versus-host disease, host rejection, altered persistence, and genome-engineering toxicity (125, 126). Their use in asthma should therefore be matched to the intended function. Allogeneic or iPSC-derived CAR-NK products may be particularly suitable for bounded depletion of well-validated pathogenic targets, where limited persistence may be advantageous in a nonmalignant disease. Experience with an iPSC-derived CD19/BCMA CAR-NK product in systemic sclerosis provides proof of principle for such platforms in immune-mediated disease, although not specifically in asthma (127). Future asthma programs should ideally minimize conditioning and prolonged alloreactivity and incorporate controllable persistence or shutdown mechanisms. Thus, rapid autologous point-of-care manufacture may better suit regulatory or tolerance-restoring strategies, whereas standardized allogeneic products may be more appropriate for short-duration cytotoxic programs.

Clinical development should avoid copying oncology endpoints. In first-in-asthma phase 1/1b studies, safety and tolerability should remain the primary objectives, with CAR-cell pharmacology, target engagement, and target-cell depletion or regulation treated as key translational endpoints; clinical efficacy should remain exploratory. Safety assessment should include pulmonary deterioration, cytokine-release and neurological toxicities where relevant, infection, cytopenias, on-target/off-tissue effects, and the need for shutdown or rescue intervention (98, 116). Exploratory efficacy outcomes may include exacerbations, systemic corticosteroid exposure, symptom control, lung function, patient-reported outcomes, and multidomain remission (128). Clinical improvement in a small early-phase study should not itself be interpreted as evidence of disease modification.

Remission, treatment withdrawal, disease modification, and immune reset should be distinguished explicitly. Remission is a clinical state and may occur while guideline-directed inhaled controller therapy continues (4, 129, 130). In early CAR studies, inhaled corticosteroid-containing therapy should therefore initially remain stable, whereas withdrawal of a maintenance biologic or tapering of maintenance oral corticosteroids should be protocolized and reported separately. “Treatment-free” should specify which treatment has been withdrawn. Disease modification should refer to benefit that persists after active CAR-mediated control has substantially waned, whereas “immune reset” should be reserved for the more specific situation in which the relevant immune compartment reconstitutes without restoration of the pathogenic state or clinical relapse (4, 12, 13). Serial assessment of CAR persistence, target-cell reconstitution, and relapse is therefore required to distinguish ongoing CAR-mediated disease control from a durable post-treatment effect.

Dose selection should be more conservative than in oncology. The objective is not maximal tolerated dose but minimum biologically effective exposure. Sentinel dosing, staggered enrollment, intensive pulmonary monitoring, and predefined criteria for pharmacological shutdown or cell elimination are appropriate for first-in-asthma studies. Lymphodepletion should be minimized or avoided if possible because it adds infection and immune-reconstitution risk that may be difficult to justify in a chronic respiratory disease. Preclinical work in immunocompetent asthma models without conventional conditioning is therefore particularly valuable (17).

First-in-human studies in asthma require a particularly stringent regulatory and ethical framework because the disease is nonmalignant and effective approved therapies are available. The acceptable risk–benefit threshold should therefore be higher than in refractory malignancy, with initial enrollment restricted to patients with substantial residual morbidity despite optimized, mechanistically appropriate treatment (4). Long-term surveillance should assess CAR-cell persistence, immune reconstitution, delayed toxicity, and secondary malignancy, with follow-up intensity and duration tailored to product-specific risks (4, 131). Informed consent should clearly address uncertain benefit, established therapeutic alternatives, potentially irreversible biological effects, and the burden of prolonged surveillance (105). Independent safety oversight, predefined stopping criteria, and rescue or cell-elimination procedures should also be incorporated into early clinical protocols.

A realistic first clinical program would be small, biomarker-enriched, and mechanistically narrow. Candidates should have severe type 2-high disease with recurrent exacerbations or persistent systemic corticosteroid dependence despite verified, adequately dosed, and adequately assessed trials of one or more mechanistically appropriate biologics. For an eosinophil-directed program, eligibility should additionally require evidence that a clinically relevant target-positive eosinophilic compartment persists despite matched anti-IL-5/IL-5Rα therapy (132); clinical nonresponse in the setting of effective eosinophil suppression would instead argue against further eosinophil-directed depletion. Patients whose residual morbidity is predominantly attributable to fixed remodeling, major comorbidities, or an alternative inflammatory pathway should be excluded from an eosinophil-directed program unless a different CAR mechanism is independently justified (105). The initial objective should be to determine whether a controllable or transient product can safely achieve target engagement and clinically meaningful disease control while guideline-directed inhaled controller therapy remains stable; withdrawal of maintenance biologics and tapering of systemic corticosteroids should be assessed separately as protocolized exploratory outcomes (76).

## 10 Translational roadmap and emerging platforms

The most important technological direction for asthma is not stronger CAR signaling but better control over where and for how long engineered cells act. Transient mRNA delivery can generate CAR activity without permanent genomic expression, and nanoparticle systems can program circulating T cells *in vivo* (133). *In vivo* CAR generation has already been used experimentally against activated fibroblasts (14). These approaches could convert a bespoke cell product into a more drug-like, repeatable intervention and are especially attractive for asthma, where a reversible first exposure is preferable to irreversible persistence.

Programmable receptor systems offer a second direction. Universal adaptor CARs can separate cell manufacture from antigen specificity, while protease-regulated and rest-inducible systems can alter receptor availability or recover function (134–136). In asthma, a universal platform could permit switching between targets as disease biology evolves, and a regulated system could provide planned treatment windows rather than continuous activity. However, platform flexibility should not substitute for biological precision. Every added component creates new immunogenicity, pharmacology, and manufacturing questions that need explicit validation.

Organ-selective delivery could further shift the therapeutic index. Nanoparticle chemistry can bias nucleic-acid delivery toward specific organs, and lipid nanoparticles have been optimized for nebulized mRNA delivery to the lung (137, 138). Regional CAR delivery has also shown clinical feasibility in pleural disease (111). These technologies suggest a future in which engineered immune activity is generated or concentrated in the respiratory tract rather than systemically. The ideal strategy may differ by target: airway-resident regulatory cells could benefit from local induction, whereas eosinophil precursors in bone marrow would require systemic access.

Multi-omic target discovery is likely to be decisive. Bulk biomarkers are insufficient for a therapy whose safety depends on cell-state specificity. Single-cell transcriptomics, spatial profiling, surface proteomics, and ligand–receptor inference can identify disease-associated states, define their anatomical relationships, and map expression in healthy tissue (99). The next step should be functional perturbation: candidate targets identified computationally must be tested to determine whether selective removal or regulation actually collapses the pathogenic network. Observational association is not enough for a durable cell therapy.

For type 2-low or non-eosinophilic asthma, target discovery should begin with molecular stratification rather than *a priori* selection of a CAR antigen. Multi-omic analysis of U-BIOPRED sputum identified distinct severe neutrophilic states, including a microbiome-associated cluster enriched for *Moraxella catarrhalis* and *Haemophilus influenzae* and another associated with IL-22 signaling (4). Single-cell analysis has also identified disease-associated epithelial alterations in non-eosinophilic asthma, including loss of CFTR-expressing airway ionocytes (4). These findings support a staged strategy integrating single-cell transcriptomics, surface-protein profiling, and spatial mapping to identify stable, surface-accessible marker combinations within pathogenic immune or structural niches. Candidate populations should then undergo normal-tissue mapping and reversible functional perturbation to establish causal relevance and safety. Only targets whose selective depletion or regulation modifies established disease without compromising host defense should progress to transient or logic-gated CAR development (4, 99, 139).

Computational engineering can also improve receptor design. Integrated transcriptomic and proteomic approaches have been used to prioritize combinatorial CAR targets, quantitative models can estimate the discriminatory value of multi-antigen recognition, and machine-learning approaches can relate signaling motif combinations to CAR phenotypes (139–141). For asthma, these methods could be used to identify surface-marker combinations that distinguish pathological immune or stromal states from homeostatic counterparts and to design circuits with activation thresholds matched to target density. Such tools are most valuable when they solve a specific biological selectivity problem rather than simply maximizing *in vitro* activity.

Existing biologics may also become sequencing or rescue tools rather than simple competitors. Anti-IL-5/IL-5Rα and related agents can reduce active inflammatory burden and, in some studies, remodeling-associated signals (142, 143). Whether pretreatment improves the safety or pharmacology of a cellular intervention is unproven and should be tested rather than assumed. Conversely, biologics could be restarted if a transient CAR intervention durably modifies one compartment but leaves another pathway active. Early translation should therefore prespecify sequencing and rescue strategies.

A staged development roadmap follows directly from the four gates. Stage 1 should use human airway single-cell, spatial, and surface-proteomic datasets to identify stable targetable states and quantify normal-tissue expression. Stage 2 should use reversible perturbation—transient CAR expression, switchable receptors, or short-lived cell platforms—to test causal target dependence in established disease models. To improve translational robustness, this stage should use a panel of complementary, reproducible models that captures established and remodeled disease, distinct inflammatory endotypes, and clinically relevant modifiers such as obesity, age/sex, respiratory infection, and prior corticosteroid or biologic exposure, rather than relying on a single acute allergic-airway model. Stage 3 should integrate target pharmacodynamics with lung trafficking, immune reconstitution, and pulmonary safety; in first-in-human studies, safety should remain primary, whereas asthma efficacy, remission, and protocolized treatment withdrawal should be exploratory. Highly persistent or multifunctional products should advance only after the target is shown to be both necessary and safely modifiable.

## 11 Discussion and perspectives

CAR-engineered cell therapy is credible as a research direction for severe asthma, but it is not a clinical category ready for broad adoption. Peer-reviewed direct asthma studies establish three addressable modes of action: localized CAR-Treg regulation, eosinophil-lineage depletion with or without parallel cytokine modulation, and allergen-focused tolerance restoration (4, 15–17). Disease-adjacent membrane-IgE engineering and patient-derived eosinophilic data provide additional mechanistic support (19, 20), while autoimmune studies show that nonmalignant immune compartments can be targeted with durable or transient CAR platforms (4, 76, 79, 80, 85). The advance is therefore not clinical asthma efficacy—which remains absent—but a sufficiently developed preclinical evidence base to ask which targets, functions, and patients justify further translation. This positioning is consistent with broader discussions of CAR immunotherapy beyond cancer and its possible extension to allergy (144, 145).

Four tensions should organize the next phase of the field. First, depth of intervention must be balanced against physiological dispensability: moving upstream from eosinophils toward memory or antibody-producing reservoirs may increase durability while increasing the consequences of target error. Second, persistence must be balanced against reversibility; long-lived cells may sustain control but also prolong on-target/off-tissue effects, making transient expression, pharmacological control, or switchable systems particularly attractive in a nonmalignant disease (146). Third, systemic access must be balanced against anatomical precision because airway mucosa, lung interstitium, lymphoid tissue, and bone marrow represent different target geographies. Fourth, engineering complexity must solve a defined biological problem rather than merely add technical sophistication.

The concentration of current evidence in type 2-high disease is an important limitation. It should not be interpreted as evidence that type 2-low severe asthma is intrinsically unsuitable for engineered-cell therapy. Rather, neutrophilic, Th17-associated, inflammasome-related, interferon-linked, metabolic, and paucigranulocytic states currently lack a validated surface target that combines causal importance, sufficient stability, anatomical accessibility, and physiological dispensability (139, 140). For type 2-low disease, the priority should therefore be target discovery rather than premature CAR construction. A practical strategy is to first resolve molecularly distinct neutrophilic or paucigranulocytic states and then identify stable, surface-accessible marker combinations within their disease-maintaining cellular niches using integrated single-cell, spatial, and surface-proteomic profiling. Candidate populations should subsequently undergo reversible functional perturbation to establish causal dependence before CAR engineering is considered (4, 99).

## 12 Limitations of the evidence base and of this review

Several limitations should temper interpretation of the proposed framework. Direct asthma evidence remains small and predominantly preclinical, and allergic-airway models do not reproduce the full heterogeneity, comorbidity, remodeling burden, or treatment history of severe human asthma. Important arguments about immune reset, transient CAR pharmacology, safety control, and alternative cellular chassis are extrapolated from autoimmune disease, oncology, or non-asthma fibrosis. This is a narrative synthesis without preregistered study selection, formal risk-of-bias assessment, or quantitative evidence grading, and the domain-level judgments in **Table 2** are qualitative decision aids rather than validated scores. The field is also moving rapidly; target appraisal and platform readiness may change as new human airway datasets and asthma-specific engineering studies emerge. The framework should therefore be used as a decision aid for translational prioritization, not as evidence that any specific CAR strategy is clinically ready.

A major preclinical priority is the development of robust and reproducible animal models that better reflect the heterogeneity of severe human asthma. Reliance on a single acute allergen-driven model is insufficient, and candidate CAR strategies should be evaluated across complementary models incorporating established and remodeled disease, relevant inflammatory endotypes, and clinically important modifiers such as comorbidities, infection, and prior treatment exposure. Standardized protocols, prespecified endpoints, blinded assessment, and independent replication will also be important to distinguish reproducible therapeutic effects from model-specific findings. Such studies should integrate efficacy with CAR-specific pharmacodynamic and safety measures, including pulmonary trafficking, persistence, target distribution, and on-target/off-tissue effects.

Two limitations within the direct asthma evidence tier deserve particular emphasis. First, the CEA-directed CAR-Treg study (15) depended on CEA-transgenic recipients to provide a pulmonary antigen for CAR engagement. It therefore establishes the principle that tissue-directed CAR activation can enhance local Treg-mediated suppression, but its dependence on an experimentally introduced target constrains extrapolation to naturally occurring human asthma. Second, although the Bet v1-specific CAlleR-Treg study (18) included both prophylactic and treatment schedules, follow-up after cell transfer was limited to approximately one to two weeks. The available data therefore support short-term efficacy but are insufficient to establish durable CAR-Treg persistence or durable disease modification.

The comparator determines the acceptable risk. An important theoretical disadvantage of durable cytotoxic CAR interventions, relative to established biologics, is that prolonged engineered-cell activity and previously induced lineage depletion may not be promptly reversible (4). Longer-term studies must establish whether targeted cell populations and their protective functions recover within a clinically acceptable period after CAR activity ceases (77). Modern biologics can substantially reduce exacerbations and corticosteroid exposure, some patients achieve multidomain remission, and treatment burden continues to fall with long-interval agents (147). A CAR product therefore needs more than another route to cytokine suppression or less frequent dosing. Its strongest value proposition is a finite intervention that removes or reprograms a disease-maintaining cellular reservoir and permits sustained disease control after active CAR-mediated effects have waned, with reduced or eliminated dependence on maintenance biologic therapy and systemic corticosteroids. Broad CD19 depletion is informative as proof of principle but difficult to justify by default; eosinophil-lineage targeting, allergen-specific regulation, or other biomarker-defined strategies provide more plausible starting points.

The translational sequence should remain conservative. Human airway datasets should first establish target distribution and normal-tissue safety; reversible platforms should then test causal dependence in established, immunocompetent disease models; and persistence or multifunctionality should be increased only if the target is validated and insufficient exposure is the limiting factor. Pharmacodynamic readouts should include target engagement, pulmonary trafficking, immune reconstitution, and the relationship between target return and clinical relapse. For a chronic disease, the most persuasive evidence of disease modification is sustained improvement after engineered activity has been reduced or withdrawn, not prolonged CAR-cell detectability.

For most patients with asthma, CAR-engineered therapy will not improve the risk–benefit balance over biologics. The plausible niche is biologic-refractory disease in which a disease-maintaining cellular compartment is demonstrable, targetable, and sufficiently dispensable—or safely regulatable—to justify intervention. Candidate programs should therefore be judged by three linked decisions—the right target, the right function, and the right patient—under a fourth requirement: controllability is part of efficacy.
